# Receptor Tyrosine Kinases as Candidate Prognostic Biomarkers and Therapeutic Targets in Meningioma

**DOI:** 10.3390/ijms222111352

**Published:** 2021-10-21

**Authors:** Rafael Roesler, Barbara Kunzler Souza, Gustavo R. Isolan

**Affiliations:** 1 Department of Pharmacology, Institute for Basic Health Sciences, Federal University of Rio Grande do Sul, Porto Alegre 90050-170, Brazil; rafaelroesler@hcpa.edu.br; 2 Experimental Research Center, Cancer and Neurobiology Laboratory, Clinical Hospital (CPE-HCPA), Federal University of Rio Grande do Sul, Porto Alegre 90035-003, Brazil; barbara@epigenica.com.br; 3 Epigenica Biosciences, Canoas 92035-000, Brazil; 4 Graduate Program in Surgery, Mackenzie Evangelical University of Paraná (FEMPAR), Curitiba 80730-000, Brazil; 5 The Center for Advanced Neurology and Neurosurgery (CEANNE)-Brazil, Porto Alegre 90050-170, Brazil

**Keywords:** epidermal growth factor receptor, ErbB, biomarker, meningioma, intracranial tumor

## Abstract

Meningioma (MGM) is the most common type of intracranial tumor in adults. The validation of novel prognostic biomarkers to better inform tumor stratification and clinical prognosis is urgently needed. Many molecular and cellular alterations have been described in MGM tumors over the past few years, providing a rational basis for the identification of biomarkers and therapeutic targets. The role of receptor tyrosine kinases (RTKs) as oncogenes, including those of the ErbB family of receptors, has been well established in several cancer types. Here, we review histological, molecular, and clinical evidence suggesting that RTKs, including the epidermal growth factor receptor (EGFR, ErbB1), as well as other members of the ErbB family, may be useful as biomarkers and therapeutic targets in MGM.

## 1. Introduction

Meningiomas (MGMs) constitute the most common type of primary intracranial tumor in adults, accounting for a little over a third of all intracranial neoplasms. An MGM is defined as a tumor emerging from the meninges, which consist of the dura mater, arachnoid, and pia mater that envelope the brain and spinal cord. Most MGM tumors are benign, but they can present grades of dedifferentiation from grade I to grade III (anaplastic/malignant) that are associated with aggressiveness. MGMs occur more commonly in adult females than in males, except for higher grades, as well as in elderly patients, but are rarer in children and adolescents. Advancements in imaging methods have considerably improved our ability to diagnose MGM, thus, the diagnosis rate has increased. MGM tumors typically grow slowly and are not infiltrative. Common symptoms include headaches secondary to increased intracranial pressure, focal neurological deficits, and seizures either involving the tumor tissue itself or caused by mass effect. Currently, MGM is classified into fifteen histologic subtypes across three grades of malignancy by a World Health Organization (WHO) grading system intended to reflect recurrence rate and prognosis. Thus, grade I benign, grade II atypical, and grade III anaplastic MGMs are further divided into 15 subtypes, among which meningothelial, fibroblastic, and transitional MGM are the most common [1,2,3]. In addition to histopathological analysis, positron emission tomography (PET) imaging has contributed to the distinction between low- and high-grade MGMs [4]. Additionally, in magnetic resonance imaging (MRI), high apparent diffusion coefficient (ADC) is a parameter that may enable the distinction of MGM tumor regions enriched in proliferating cells that display developmental gene expression programs [5].

Moreover, other classification methods have been more recently proposed, based on molecular markers such as DNA methylation profiles and other genetic and epigenetic parameters. Mutations of the *TERT*, *BAP1*, *DMD*, and *CDKN2A/B* genes have been proposed to aid in risk stratification. An additional level of classification based on epigenetics has been proposed in which MGM tumors are classified into group A, further subdivided into MC ben-1, ben-2, ben-3, and MC int-A, and group B, subdivided into MC int-B and MC mal. The potential role of histone methylation patterns and recurrent mutations in genes that encode components of epigenetic regulation has been explored, an example being the association between the absence of histone 3 lysine 27 trimethylation (H3K27me3) as indicative of poor prognosis [6,7].

MGM biology reveals biological parallels between meningeal embryology and the arising of tumors. MGM tumorigenesis and meningeal development during embryogenesis are related regarding the likely cells of origin, the role of stem cells, and signaling mechanisms that program development. Arachnoid cap cells, and, more specifically, prostaglandin D2 synthase (PGDS)-positive arachnoid cells, are the most likely MGM cells of origin [8,9,10], and the presence of a cancer stem cell compartment has also been proposed. As in other types of cancer, cancer stem cells within MGM tumors may represent a subpopulation promoting recurrence, resistance to treatment, and metastasis, and efforts are currently being undertaken to characterize MGM stem cell markers [11]. The five-year survival for WHO grade I MGM is over 80%, but patients with anaplastic MGM show greatly reduced survival. The standard treatment in surgically accessible tumors is total surgical resection, which is capable of curing up to 80% of MGM cases. Radiotherapy is now the gold standard in patients with grade III tumors, used in atypical and anaplastic MGMs, which often show higher recurrence rates, intense invasiveness, and poor prognosis, and in recurrent tumors and surgically inaccessible MGMs. Adjuvant chemotherapy has not, so far, been shown as effective, and its use is limited to high-grade aggressive and relapsing tumors, and there are no established standards for the use of systemic therapies. Some chemotherapeutics, such as the transcription inhibitor trabectedin and hydroxyurea (hydroxycarbamide), which inhibit ribonucleotide reductase, thus reducing the synthesis of deoxyribonucleotides, have been tested with promising results in preclinical and clinical models of MGM [12,13,14,15].

## 2. Molecular Changes and Novel Molecularly Targeted Therapeutic Strategies

Several aspects of the genetic and cellular bases underlying MGM have been unraveled and provide novel opportunities for the development of targeted treatments. Next-generation sequencing revealed recurrent somatic mutations in the neurofibromatosis 2 (*NF2*), TNF receptor associated factor 7 (*TRAF7*), Krüppel-like factor 4 (*KLF4*), AKT serine/threonine kinase 1 (*AKT1*), smoothened (*SMO*), and phosphatidylinositol-4,5-bisphosphate 3-kinase catalytic subunit alpha (*PIK3CA*) genes, which are collectively found in around 80% of sporadic MGMs and are associated with tumor location, histologic subtype, and clinical outcome [6,7,10,16,17,18]. The most common chromosomal abnormality in MGM is found in chromosome 22 and occurs in 40–70% of grade I tumors. Beyond the loss of chromosome 22, few other chromosomal abnormalities have been observed in benign MGM [10,19]. All or part of chromosome 22 is deleted, and most deletions are found in the *NF2* region, suggesting a role for mutated *NF2* as an oncogene in MGM pathogenesis [10,20]. Loss of chromosome 1 is the second most common deletion site in MGM, mostly observed in atypical and anaplastic tumors [21]. Activating mutations in the promoter of the telomerase reverse transcriptase (*TERT*) gene are associated with higher recurrence, faster progression, and poorer overall survival [22]. Genomic deletion or reduced protein expression of dystrophin (*DMD*) gene are found in around one third of MGM patients and associated with poorer overall survival. MGMs showing deficient expression of the tumor suppressor gene breast cancer (BRCA)1-associated protein-1 (*BAP1*) are more aggressive and lead to poor prognosis [10,23].

Inhibition of receptor tyrosine kinases (RTKs) has been experimentally explored as a basis to reduce tumor growth in MGM models. High levels of phosphorylated epidermal growth factor receptor (pEGFR) are found in both lysates of MGM tumors and MGM cells compared to non-tumoral control tissues. Signaling mediated by EGFR mediates aberrant STAT1 activation, and EGFR inhibition impairs cell proliferation and reduces the levels of cyclin D1, phosphorylated AKT, and phosphorylated extracellular signal-regulated kinase (ERK)1/2 [24]. Inhibition of platelet-derived growth factor receptor (PDGFR)-mediated signaling by RTK inhibitors sorafenib and regorafenib inhibits the proliferation of IOMM-Lee MGM cells, as well as the growth of experimental IOMM-Lee MGM tumors in vivo in mice inoculated with IOMM-Lee MGM cells into the subarachnoidal space, through PDGFR downregulation and inhibition of expression and phosphorylation of p44/42 ERK [25]. A few cases of tumor responses to the multiple RTK inhibitor sunitinib have been reported in patients with grade II and III MGMs [26,27]. The subjects in one trial were patients with recurrent or progressive atypical and anaplastic MGM tumors, heavily pretreated and refractory to treatments. That study was the first prospective trial showing an effective treatment in patients with aggressive MGM. Treatment with sunitinib resulted in 42% of patients alive and progression-free at 6 months. However, considerable toxicity was observed [26]. When patients with recurrent MGM tumors, refractory to surgery and radiation, were treated with the oral multi-RTK inhibitor PTK787/ZK 222584 (PTK787), this showed that those with grade II MGM had progression-free survival at 6 months of 64.3%, a median progression-free survival of 6.5 months, and an overall survival of 26.0 months; patients with grade III MGM had a progression-free survival at 6 months of 37.5%, a median progression-free survival of 3.6 months, and overall survival of 23 months [28]. A small retrospective study of 18 patients with recurrent MGM, nine among them with PDGFR-positive tumors, suggested that treatment with the PDGFR inhibitor imatinib mesylate may be a well-tolerated therapeutic option capable of stabilizing disease in a group of patients preselected on the basis of tumor positivity for PDGR [29]. Table 1 presents a summary of selected experimental and clinical studies providing evidence for RTKs as potential therapeutic targets in MGM.

In terms of intracellular signaling and angiogenesis pathways, increased activity of the phosphoinositide 3-kinase (PI3K)/AKT/mechanistic target of rapamycin (mTOR) cascade has been reported in MGM, and treatment with mTOR inhibitors reduces experimental MGM growth [30], and mTOR inhibition impairs neuregulin 1-ERBB3 autocrine signaling in *NF2*-deficient cellular models of MGM [31]. In addition, a phase 2 clinical study of the mTOR inhibitor everolimus in MGM has provided satisfactory early results [32].

### Potential Role for Anti-Angiogenic, Hormone, and Immune-Based Therapeutical Modalities in MGM

Expression levels of the vascular endothelial growth factor (VEGF) in atypical and anaplastic MGM are higher than in benign MGM [33]. Small clinical trials have provided early evidence suggesting that treatment with the anti-VEGF monoclonal antibody bevacizumab or the small molecule VEGF and PDGF receptor inhibitor sunitinib may increase progression-free survival in patients with MGM [26,34,35,36,37,38]. A case study of two radiographically diagnosed intracranial MGM tumors in a patient with concurrent thyroid carcinoma described tumor regression after treatment with cabozantinib, a small molecule RTK inhibitor with potent activity against the VEGF receptor type 2 (VEGFR2) [39]. In the light of increased expression of the programmed death-ligand receptor (PD-L1) in MGM [40], immunotherapy with nivolumab and pembrolizumab is currently under evaluation in phase 2 clinical trials of MGM [10]. MGM cells express progesterone receptor (PR), and the potential of the PR antagonist mifepristone in MGM treatment has been explored; however, the currently available clinical results still do not allow the conclusion that any meaningful beneficial effect exists [41,42,43]. In summary, despite current exploration of cellular components including some RTKs and intracellular signaling pathways, in addition to hormonal modulation, angiogenic processes, and immune responses, as therapeutic targets, to date there is no strong evidence supporting the benefit of pharmacological interventions for MGM patients.

## 3. Current and Candidate Biomarkers in MGM

Many radiological, plasmatic, histological, and molecular prognostic markers have been put forward to help to stratify MGMs. However, to date there are no clinically validated biomarkers to help inform the determination of tumor grade and clinical prognosis [21,44]. Investigation of gene expression features of progressing, recurrent, or grade III MGM tumors revealed that notably aggressive tumor subsets share a substantial group of differentially expressed genes, in addition to identifying genes separating non-recurring from recurrent and malignant grade I or grade II tumors. Moreover, a significant association of a subset of genes with progression-free survival was shown [45].

Proteomic approaches have aided in the effort to validate candidate biomarkers [46]. For example, bioinformatics combined with the analysis of protein content profile applied to tumor and blood samples from MGM patients has pointed to proteins including serpin peptidase inhibitor alpha 1, ceruloplasmin, hemopexin, albumin, C3, apolipoprotein, haptoglobin, amyloid-P-component serum, and alpha-1-beta-glycoprotein as potential prognostic markers [47]. A small study aimed at identifying MGM-specific proteins in the cerebrospinal fluid (CSF) from four MGM patients and four patients with a non-brain tumor found increased levels of apolipoprotein E (ApoE), apolipoprotein J precursor (ApoJ), and alpha-1-antitrypsin (AAT), and reduced levels of prostaglandin D2 synthase 21 kDa (PTGDS), transthyretin precursor (TTR), and β-2-microglobulin precursor (β2M) [48]. Additionally, using a proteomic strategy, one group has put forward retinoblastoma associated protein-1 (RB1) as a critical marker to identify grade I MGM tumors with high risk for recurrence [49].

Other studies quantifying protein content via multiple techniques have aided in the identification of possible protein MGM biomarkers. Semiquantitative analysis of nuclear expressions of karyopherin a2 and chromosome region maintenance protein 1, members of the karyopherin protein family that comprise nucleocytoplasmic shuttling receptors importins and exportins, revealed that expression of these proteins correlated significantly with MGM histological grade and predicted tumor recurrence [50]. Change in securin (*PTTG1*) gene expression, which prevents sister chromatid separation, and alteration of leptin receptor (*LEPR*) gene expression, were associated with MGM malignancy [44]. The somatostatin receptor 2A (SST2A) has been proposed as a good immunostain target due to its high sensitivity [51]. Experiments using protein separation by two-dimensional gel electrophoresis and the identification of candidate biomarkers by liquid chromatography–mass spectrometry identified seven candidate protein biomarkers, which were capable of differentiating between aggressive and benign WHO grade I MGMs [52]. A study focusing specifically on stem cell-related protein markers revealed differential expression of the G protein-coupled receptor Frizzled 9 (cluster of differentiation 349, CD349) and glial fibrillary acidic protein (GFAP) in grade II/III compared with grade I MGM. GFAP expression correlated with the stem cell markers CD133, stage specific embryo antigen 4 (SSEA4), and vimentin in cell populations enriched in grade II/III tumors [53]. The measurement of protein serum markers has revealed significant increases in amphiregulin (AREG), EGF, HB-EGF, and caspase 3 in patients with MGM of different grades, helping to establish an MGM protein signature in the blood [54].

Epigenetic changes have been put forward as prognostic markers in MGM [2]. Enhancer of Zeste homolog-2 (EZH2) and trimethyl histone-3 (H3K27me3), which mediate histone modifications related to chromatin state, have been examined. Low histological levels of H3K27me3 were found by a systematic review as a marker that may aid the differentiation between grade I and grade II tumors [44]. Immunohistochemical analysis of 149 cases of MGM tumors grade I (*n* = 102) or grade II (*n* = 47) has indicated that positivity for EZH2 and negativity for H3K27me3 are associated with higher tumor cell proliferation and are significantly more common in grade II MGM compared to grade I tumors. Expression of EZH2 and loss of H3K27me3 are significantly associated with shorter progression-free survival. DNA methyltransferases (DNMT)-1, -3A, and -3B, which control DNA methylation, are found in most tumors of either grade, with higher DNMT-1 content in grade II MGMs [55].

Several mutations found in MGM can drive epigenetic alterations, particularly methylation profiles [21]. These include the inactivation of genes encoding subunits of the SWI/SNF chromatin remodeling complexes [56,57] and loss of the retinoblastoma protein-interacting zinc-finger (*RIZ*) gene [58]. The loss of *RIZ* associates with tumor progression, being inversely correlated with MGM grade [57]. Hypermethylation accompanied by loss of gene expression of WNK lysine deficient protein kinase 2 (*WNK2*), a negative regulator of cell proliferation, is found in 83% of grade II and 71% of grade III tumors [59]. Epigenetic characterization of MGM has also provided unique DNA methylation profiles that allow the segregation of all MGM types, across grades, from other skull tumors, and these classifications can predict progression-free survival with higher accuracy compared to the WHO grade alone [21,60]. The extent of methylation occurring in a set of five homeobox genes (*HOXA6*, *HOXA9*, *PENK*, *UPK3A*, and *IGF2bP1*) may predict MGM recurrence [61]. An analysis of microRNA (miRNA) levels in MGM tumors of different grades and in serum found that expression of the miR-497~195 cluster decreases with increased malignancy. Overexpression of cyclin D1 is associated with downregulation of the miR-497~195 cluster. The transcription factor GATA binding protein 4 (GATA-4), which is upregulated in malignant MGM, upregulates cyclin D1, thus controlling miR-497~195 cluster expression and stimulating cell viability. Levels of miR-497 are lower in serum exosomes derived from patients with high-grade MGM compared to benign MGM. These data suggest miR-497 as a potential non-invasive biomarker for malignant MGM [62].

The top 19 differentially expressed miRNAs were subject to validation by reverse transcription-quantitative polymerase chain reaction (RT-qPCR) using total RNA of fifteen MGM patients’ tumor samples and five meninges control samples. Tumor suppressor miRNAs miR-218 and miR-34a appeared to be increased relative to normal controls. By contrast, miR-143, miR-193b, miR-451, and miR-21 appeared to be decreased. A total of ten putative mRNA targets were selected to be tested by RT-qPCR, and four of them were found to be significantly differentially expressed relative to normal controls at a threshold of *p*-value < 0.05. *PTEN*, E-cadherin (*CDH1*), and *p63* were upregulated, whereas *RUNX1T1* was downregulated. Nuclear Cyclin D1 expression was found to be present as a strong or moderate signal among all studied MGMs, regardless of being MGMs of grade I or II. Validation in a larger number of patients is needed [63].

Another study suggested a panel of six serum miRNAs as potential biomarkers. Specifically, serum levels of miR-106a-5p, miR-219-5p, miR-375, and miR-409-3p are significantly increased in MGM patients, whereas serum levels of miR-197 and miR-224 are reduced. Levels of the four increased miRNAs significantly decrease after surgical MGM removal, whereas the two reduced miRNAs increase. In addition, expression levels of miR-219-5p are positively associated with tumor clinical stage. Moreover, high expression of miR-409-3p and low expression of miR-224 are associated with recurrence [64]. Together, these findings suggest that the combined analyses of genomic, epigenetic, and protein biomarkers may enable the development of a new panel to help predict aggressive MGM with high rates of progression and recurrence.

## 4. RTKs as Candidate Prognostic Biomarkers in MGM

The role of abnormal RTK signaling in oncogenesis and tumor progression is well established. For example, overexpression and activating mutations of genes encoding members of the ErbB receptor family (for example, EGFR or ErbB1 in non-small-cell lung cancer and colorectal cancer, and ErbB2, also called HER2, in breast cancer) are used to identify subgroups of tumors responsive to small molecule agents (e.g., gefitinib, erlotinib) or monoclonal antibodies (e.g., cetuximab, trastuzumab) that specifically target ErbB receptors [65,66,67]. Mutations and overexpression of RTKs can also be used as predictive markers of response to targeted therapies. For example, *EGFR* gene copy number has also been put forward as a candidate biomarker for predicting treatment response to EGFR inhibitors in patients with advanced non-small-cell lung cancer and colorectal cancer [68]. As an illustration in brain tumors, the *EGFR* gene is among the most frequently altered oncogene in glioblastoma (GBM), with 57% of tumors showing amplification, mutation, rearrangement, or altered splicing [69], and EGFR has been put forward as a prognostic biomarker in GBM [70], although conflicting results have been reported [71].

Emerging evidence suggests a potential role for RTKs as biomarkers in MGM. A study using tissue microarrays obtained from a set of 186 MGM tumors analyzed by immunohistochemistry with antibodies targeting intracellular and extracellular domains of EGFR and pEGFR revealed that EGFR is overexpressed and activated in most human MGM cases. Remarkably, survival or recurrence was significantly decreased in association with high staining of the EGFR extracellular domain [72]. Another immunohistochemical study of 113 MGM specimens from 89 patients indicated that EGFR expression may be higher in benign MGM tumors. Thus, a staining percentage score for EGFR expression was high in benign and atypical tumors but low in all malignant tumor samples evaluated [73]. The examination of 115 MGM tumors via next-generation sequencing, immunohistochemistry, and fluorescent and chromogenic in situ hybridization confirmed expression of EGFR in 93% of samples [74]. Overexpression and constitutive phosphorylation of EGFR-signal transducer and activator of transcription 1 (STAT1) was found in a set of 131 MGMs of different grades and locations by Western blots, qPCR, and immunocytochemistry. In addition, high expression levels of pEGFR were found both in MGM specimens and primary cell cultures [24]. A study using high throughput tissue microarray immunohistochemistry (TMA-IHC) that included 41 MGMs of various grades as well as two subsets of atypical MGMs found that EGFR is differentially expressed in symptomatic, surgically resected MGMs versus incidental MGMs, and PDGFRβ helps to distinguish anaplastic MGMs from hemangiopericytomas [75]. Northern blot analysis revealed expression of EGFR mRNA in nine of eleven (82%) primary MGM tumors, and immunocytochemistry confirmed strong positivity at the protein level. In contrast, no EGFR expression was found in samples of non-neoplasical meninges [76]. Another study detected EGFR by immunoblot in six out of nine MGMs (67%) and by immunohistochemistry in 13 of 19 (68%) MGMs, but not in normal leptomeningeal cells [77]. Importantly, analysis using immunohistochemistry and gene amplification by fluorescence in situ hybridization (FISH) showed that progression from benign to atypical or anaplastic MGMs associates with an increase in EGFR protein content, so that EGFR immunostaining directly correlates to tumor grade. However, EGFR expression was not associated with overall survival or recurrence-free survival. These findings indicate that EGFR may be a marker of tumor progression but not a prognostic marker of patient outcome [78].

A study examining 186 primary MGMs found that two members of the ErbB RTK family, HER3 and HER4, were highly expressed in most tumor samples of all grades, both in the cytoplasm and cell membrane, as well as in the nucleus for HER4. In contrast, non-neoplastic meningeal tissue was not immunoreactive, suggesting a potential diagnostic marker [79]. An immunohistochemical analysis found HER2 expression in 45% of 72 MGM tumors, being 55% grade II/III, and 38.5% of grade I. No significant differences were observed in HER2 expression between grade I and grade II/III MGM, primary and recurrent tumors, or males and females [80]. Analysis of 26 MGM samples by RT-qPCR found that mRNAs for EGFR, HER2, and HER4 were expressed in most tumors, and high HER2 content was shown by immunohistochemistry [81]. In addition, in a set of 35 MGM tumors, five atypical/anaplastic MGMs and five classic MGMs expressed HER2 protein, which was considered an overexpression in comparison with normal meninges. There was an increase in HER2 gene copy number in four of ten HER2-positive MGMs, and the rate of tumor recurrence was significantly higher in MGMs showing HER2 overexpression [82]. Moreover, in a study of 186 MGM tumors of different grades, most of which were analyzed with tissue microarrays, immunohistochemistry, and FISH, the content of activated HER2 receptors was significantly correlated with an increased risk for recurrence or death, in the absence of gene amplification or HER2 expression in normal meninges [83].

**Figure 1:**
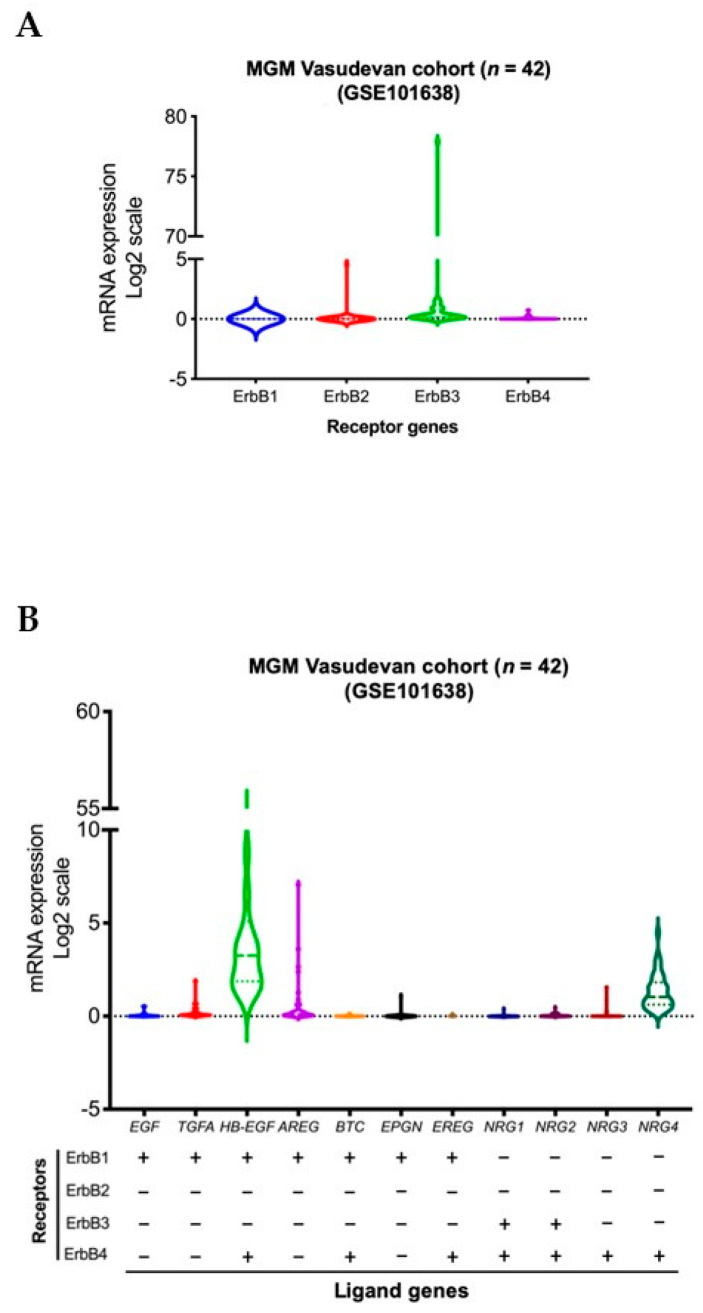
Transcript levels of members of the ErbB RTK family and their ligands in aggressive MGM, *n* = 42 MGM samples [84]. Expressions of genes for ErbB receptors (**A**) and ligands (**B**) across all samples are presented in violin format as log2-transformed signal intensity.

We conducted a violin plot analysis of data sets derived from 42 aggressive MGM tumor samples from patients in a previously published patient cohort [84]. Although the analysis reveals overall similar distribution patterns of gene expressions for different ErbB receptor family members, higher expression levels of *ErbB2* (HER2) and *ErbB3* (HER3) genes were observed in MGM patients compared to all other members of the ErbB receptor family. High levels of expression of *TGFA*, *AREG*, *EPGN* (ErbB1 or EGFR receptor ligands), and *NRG3* (ErbB4 or HER4 receptor ligand) were observed in a lower density of MGM patients. In contrast, high levels of *HB-EGF*, a ligand of EGFR and HER4, as well as *NRG4*, a ligand of HER4, were found in a higher density of patients (Figure 1).

Immunohistochemical analysis of several RTKs (VEGFR1/2/3, PDGFRα/β and c-Kit) in a set of 81 MGMs from 74 patients showed that twenty-nine grade I (45%), ten grade II (77%), and four grade III (100%) tumors were VEGFR2-positive, and VEGFR2 expression was significantly correlated with tumor grade [85]. The proto-oncogene *KIT*, which encodes the RTK KIT (cluster of differentiation 117, CD117; mast/stem cell growth factor receptor, SCFR), was robustly expressed in about 20% of MGMs, likely through upregulation of *KIT* transcription rather than gene amplification, in a study on tumor samples collected from 34 patients [86]. Another immunohistochemical study of benign MGM tumors with (*n* = 17) or without (*n* = 25) recurrence showed that coexpression of the RTK cMET and hepatocyte growth factor/scatter factor (HGF/SF) significantly associates with cell proliferation index and recurrence [87]. Table 2 presents a summary of selected studies providing evidence for RTKs as potential biomarkers in MGM.

## 5. Concluding Remarks

To date, there are no clinically effective molecularly targeted therapies to treat patients with aggressive MGM. Cell surface receptors are attractive targets that have led to several successful therapies currently being used in the clinical setting because they are more druggable molecules, compared, for example, to gene mutations, which cannot be directly corrected with drugs. As seen above, signaling by RTKs, and particularly EGFR, can be amplified in MGMs, and these tumors can respond to RTK inhibition. Future research should keep investigating the potential of targeting RTKs to treat MGM, both at the experimental and clinical trial levels.

Another aspect, which is the main focus of the current review, is the usefulness of RTKs as biomarkers in MGM. Validating clinically useful prognostic markers remains a major challenge in neuro-oncology. Ideally, biomarker validation is based on meta-analyses of molecular pathology studies using large numbers of tumors associated with clinical data. Guidelines exist that recommend methodologies, procedures for tumor collection and preparation, protocols, and reagents to be used for biomarker determination. Quality assessment and assurance programs are used for the continuous monitoring of the efficacy of biomarker use for clinical applications [88]. Additional challenges are presented by the discovery and validation of possible plasmatic biomarkers for central nervous system cancers, which would not depend on surgical removal of the tumors and could thus be used, for example, to continuously monitor treatment results and recurrence. The use of receptors in tumors as biomarkers can advance significantly in the near future thanks to the use of molecular imaging technologies, including optical imaging, MRI, single photon emission computed tomography, and PET, which can enable the visualization of RTKs in vivo [89].

In the example of EGFR as a biomarker in another brain tumor type, GBM, its characterization was based on the retrospective analyses of relationships between treatment outcomes and *EGFR* gene expression in 87 newly diagnosed patients with GBM who were enrolled in clinical trials. Southern blots and immunohistochemistry were the techniques used, leading to the observation of *EGFR* amplification in 46% of GBM tumors, with overexpression of the EGFR protein in 97.5% of the tumors displaying gene amplification. In contrast, almost 98% of patients with no *EGFR* amplification showed no EGFR overexpression. The authors then confirmed a close, statistically significant correlation between *EGFR* amplification and EGFR overexpression, and discriminated between tumors harboring wild-type versus mutated EGFR. Finally, multivariate analysis established *EGFR* amplification as an independent and significant predictor marker for poorer overall survival. This GBM study illustrates a strategy that can be used as a basis for groups aiming to better characterize the expression of RTK receptors as possible biomarkers in MGM.

The availability of accurate preoperative biomarkers in MGM patients would improve the pre-surgical assessment of these tumors, their grade, and clinical prognosis, and help direct treatment decisions [44]. Advances in the identification of biomarkers in liquid biopsies using samples of cerebrospinal fluid and blood should enable the development of less invasive diagnostic and prognostic methods that will also allow the monitoring of treatment efficacy during disease [90]. Although appropriate biomarkers for routine clinical use in the prognostic evaluation of patients with MGM remain to be characterized and will involve a complex process of validation, the findings reviewed here indicate that RTKs, particularly EGFR and other members of the ErbB family, as well as HB-EGF and NRG4 (ligands of ErbB1 and ErbB4, respectively), should be further investigated as biomarkers that are potentially capable of aiding in the early detection and determination of tumor grade and prediction of the clinical outcome upon investigation of surgically removed MGM tumors.

## Figures and Tables

**Table 1:** Summary of selected experimental and clinical studies presenting evidence for RTKs as therapeutic targets in MGM.

Experimental Model	Main Findings	References
MGM tumor specimens and primary cell culture	High levels of pEGFR are found in both MGM tumor lysates and MGM cells. Signaling by EGFR mediates aberrant STAT1 activation, and EGFR inhibition impairs cell proliferation and reduces the levels of cyclin D1, phosphorylated AKT, and phosphorylated extracellular signal-regulated kinase (ERK)1/2	[24]
Cultured IOMM-Lee MGM cells	RTK inhibitors sorafenib and regorafenib impair PDGF receptor (PDGFR)-mediated signaling and inhibit MGM cell proliferation through PDGFR downregulation and inhibition of p44/42 ERK	[25]
Orthotropic xenograft model in mice that received bilateral infusions of IOMM-Lee MGM cells into the subarachnoidal space	Treatment for 5 days a week with regorafenib inhibits intracranial MGM cell growth and cell growth and increases survival time of treated mice. In contrast, the group treated with sorafenib showed no statistically significant benefit in survival compared to controls	[25]
Thirty-six patients with either histologically proven MGM, hemangiopericytoma, hemangioblastoma, or radiographic features of a surgically inaccessible MGM, and recurrence despite radiotherapy	Treatment with sunitinib 50 mg daily for days 1–28 of 42 (one cycle), until disease progression or intolerable toxicity, resulted in 42% of patients being alive and progression-free at 6 months. Considerable toxicity was observed	[25]
Thirty-nine-year-old woman who had undergone surgeries and courses of radiotherapy over 11 years for recurrent cranial and spinal MGM.	Treatment with sunitinib resulted in a radiographic response with marked reduction in tumor volume and reduction in brainstem vasogenic edema	[27]
Patients with recurrent MGM tumors refractory to surgery and radiation	Treatment with the multi-RTK inhibitor PTK787/ZK 222584 (PTK787) led to a progression-free survival at 6 months of 64.3%, a median progression-free survival of 6.5 months, and an overall survival of 26.0 months in patients with grade II MGM, and a progression-free survival at 6 months of 37.5%, median progression-free survival of 3.6 months, and overall survival of 23 months in patients with grade III MGM	[28]
Eighteen patients with recurrent MGM	A retrospective analysis of 9 patients with PDGFR-positive MGM tumors treated with imatinib showed that 7 patients had stable disease and 2 patients had progressed at the first scan after three months. No complete or partial responses were observed. Median progression-free survival was 16 months	[29]

**Table 2:** Summary of selected studies presenting evidence for RTKs as biomarkers in MGM.

Experimental Model	Main Findings	References
MGM specimens and primary cell cultures obtained from 36 tumors	High levels of pEGFR expression across tumor samples and cultured cells	[24]
Set of 186 archived primary MGM tumors	Tissue microarrays obtained from the set of tumors and analyzed by immunohistochemistry show EGFR overexpression and activation Reduced survival and recurrence in association with high staining of the EGFR extracellular domain	[72]
Set of 113 MGM specimens from 89 patients	Benign and atypical MGM tumors show intermediate to marked staining percentage score for EGFR expression, whereas all the malignant MGM samples show low staining percentage score	[73]
Set of 115 MGM tumor specimens	Tumor investigation with next-generation sequencing, immunohistochemistry, and fluorescent and chromogenic in situ hybridization shows EGFR expression in 93% of samples	[74]
Tissue microarray from set of 41 MGMs of various grades and two subsets of atypical MGMs	Analysis by high throughput TMA-IHC show differential EGFR expression in symptomatic, surgically resected MGMs versus incidental MGMs, whereas PDGFRβ helps distinguish anaplastic MGMs from hemangiopericytomas	[75]
Set of 115 primary MGM tumors	Analysis with Northern blot shows EGFR mRNA expression in 9 (82%) tumors Expression at the EGFR protein level is confirmed by immunocytochemistry	[74]
Set of 19 MGM tumors	EGFR expression detected by immunoblot in 6 of 9 MGM tumors (67%) Immunohistochemical analysis show EGFR in 13 of 19 (68%) tumors	[77]
Set of malignant MGM tumors that progressed or not from lower grade tumors	Immunohistochemical analysis and gene amplification by FISH show that an increased EGFR expression is associated with progression from benign to atypical or anaplastic MGM tumors EGFR expression is not associated with overall survival or recurrence-free survival	[78]
Set of 186 primary MGM tumors	High expressions of HER3 and HER4 in most tumor samples of all grades, both in the cytoplasm and cell membrane, and also in the nucleus for HER4. Absence of immunoreaction in non-neoplastic meningeal tissue	[79]
Set of 72 MGM tumor samples	Immunohistochemical analysis shows HER2 expression in 45% of samples, being 55% grade II/III, and 38.5% of grade I No differences between grade I and grade II/III MGMs, primary and recurrent tumors, or males and females	[80]
Set of 26 MGM tumor samples	The mRNA expressions of EGFR, HER2, and HER4, and high protein content of HER2 in most tumors	[81]
Set of 35 MGM tumor samples	HER2 overexpression in 5 atypical/anaplastic MGMs and 5 classic MGMs; Increased HER2 gene copy number in 4 of 10 HER2-positive MGMs; Increased tumor recurrence in patients with MGMs showing HER2 overexpression	[82]
Set of of 186 MGM tumor samples of different grades	The content of activated HER2 receptors significantly correlated with increased risk for recurrence or death	[83]
Data sets derived from 42 aggressive MGM tumors	Higher levels of expressions of *ErbB2* (HER2) and *ErbB3* (HER3) compared to all other members of the ErbB receptor family High levels of expressions of *TGFA*, *AREG*, *EPGN,* and *NRG3* in a subset of patients High levels of expressions of *HB-EGF* and *NRG4* in a higher density of MGM patients	Present paper based on data from [84]
Set of 81 MGM tumors from 74 patients	Immunohistochemical analysis reveals 29 grade I (45%), 10 grade II (77%), and 4 grade III (100%) tumors positive for VEGFR2 Expression of VEGFR2 significantly correlates with tumor grade	[85]
Thirty-four tumor specimens collected from 34 patients	High expression of *KIT* in 20.6% of MGMs, likely through upregulation of *KIT* transcription	[86]
Seventeen recurrent and 25 non-recurrent MGM tumors	Significant association of coexpression of cMET and HGF/SF with cell proliferation and recurrence	[87]
